# Enhancing Surface Plasmon Resonance Detection Using Nanostructured Au Chips

**DOI:** 10.1186/s11671-016-1760-7

**Published:** 2016-12-01

**Authors:** Ivan Indutnyi, Yuriy Ushenin, Dirk Hegemann, Marianne Vandenbossche, Victor Myn’ko, Mariia Lukaniuk, Petro Shepeliavyi, Andrii Korchovyi, Roman Khrystosenko

**Affiliations:** 1 V. Lashkaryov Institute of Semiconductor Physics, Nat. Acad. of Sci. of Ukraine, Prospect Nauky, 41, 03028 Kyiv, Ukraine; 2 Empa, Swiss Federal Laboratories for Materials Science and Technology, Lerchenfeldstrasse 5, CH-9014 St.Gallen, Switzerland

**Keywords:** Surface plasmon resonance, Biosensor, Interference lithography, Vacuum chalcogenide photoresists, 73.20.Mf, 87.85.fk, 81.16.Nd

## Abstract

The increase of the sensitivity of surface plasmon resonance (SPR) refractometers was studied experimentally by forming a periodic relief in the form of a grating with submicron period on the surface of the Au-coated chip. Periodic reliefs of different depths and spatial frequency were formed on the Au film surface using interference lithography and vacuum chalcogenide photoresists. Spatial frequencies of the grating were selected close to the conditions of Bragg reflection of plasmons for the working wavelength of the SPR refractometer and the used environment (solution of glycerol in water). It was found that the degree of refractometer sensitivity enhancement and the value of the interval of environment refractive index variation, Δ*n*, in which this enhancement is observed, depend on the depth of the grating relief. By increasing the depth of relief from 13.5 ± 2 nm to 21.0 ± 2 nm, Δ*n* decreased from 0.009 to 0.0031, whereas sensitivity increased from 110 deg./RIU (refractive index unit) for a standard chip up to 264 and 484 deg./RIU for the nanostructured chips, respectively. Finally, it was shown that the working range of the sensor can be adjusted to the refractive index of the studied environment by changing the spatial frequency of the grating, by modification of the chip surface or by rotation of the chip.

## Background

Surface plasmon resonance (SPR) systems have found wide use in recent years in sensing applications due to their advantage of high sensitivity, label-free, real-time, and rapid detection. During the last two decades, the instrument development and practical applications have made great progress, which cover a large variety of fields: biotechnology, medical diagnostics, drug screening, environmental protection, and food safety [1–5]. Most of the SPR sensors make use of the attenuated total reflection method (Kretschmann configuration) [6] to excite the surface plasmon wave. Such devices typically show refractive index sensitivities for typical angular interrogation architecture ranging between 50 and 150 deg./RIU (refractive index unit) [7, 8] and refractive index resolutions in the orders of 10^−6^–10^−7^ RIU [9]. An alternative grating-coupled SPR sensor with either wavelength or angular interrogation has been demonstrated to have a sensitivity two to three times lower than devices in the Kretschmann configuration [10].

Conventional reflection-type SPR biosensors, employing a prism, however, also present several drawbacks and usually lack the sensitivity to detect the interaction of proteins with small ligands [11]. To improve the performance of SPR sensors, sensitivity enhancement has been of tremendous interest for the sensor development. In recent years, modified SPR sensing systems, such as gold nanoparticles immobilized on a gold film [12], gold nanoparticle-embedded dielectric layer on a gold film [13], and metallic nanowires regularly patterned on a metal film [14], have been proposed theoretically and experimentally. A recent theoretical study [15, 16] has shown that by formation of a sinusoidal profile grating at the surface of the metal layer of the SPR sensor used in the Kretschmann configuration, the biosensor sensitivity may be enhanced when compared with an uncorrugated metal surface. Surface plasmons propagating in a direction perpendicular to a one-dimensional grating (i.e., along the grating vector) will experience back reflections. If the period of the grating corresponds to the Bragg condition, a bandgap will then form where no surface plasmons are permitted to propagate. The control over the position of the bandgap with respect to wavelength and angle can be accomplished through the manipulation of the period of the grating. By designing the operating point of the sensor to be near the bandgap, the sensitivity can be improved compared to the sensitivity with a flat metallic layer. However, theoretical modeling has been performed for ideal structures without surface roughness and neglecting other possible defects. Such inhomogeneities can cause scattering of plasmons and broadening of the resonance curve and thus decrease the sensor sensitivity. Therefore, the made calculations should be verified experimentally.

In a previous work, we have demonstrated experimentally the possibility to increase the sensitivity of a SPR sensor that operates in the Kretschmann configuration through the formation of a periodic surface relief on the Au chip [17]. In this paper, we present more detail of the experimental study of plasmon excitation conditions in Au films with a nano-grating surface and report changes in their optical response depending on the depth of the grating relief and direction of excitation.

## Methods

Interference (interferometric) lithography (IL) has been widely used for fabrication of defect-free periodic micro- and nanostructures over the last decade. IL is a large area fabrication technique that uses laser interference patterns for rapid formation of periodic structures such as gratings and bigratings (arrays). The total processed area depends on the beam intensity and coherence length of the laser and can be up to dozens, or even hundreds of square centimeters. The technology is much cheaper and simpler than electron beam lithography and can be used for manufacturing SPR sensor structures [18, 19]. In previous studies, we have shown that IL with the use of chalcogenide photoresist is a promising technology for the formation of one- and two-dimensional submicron periodic structures on the surface of semiconductors and dielectrics [20]. Therefore, this technology was also used here for the nanostructuring of the gold films.

The samples were prepared by successive thermal vacuum deposition of (i) a 1–3-nm thick (effective thickness) Cr adhesive layer, (ii) a layer of metal (Au) with a thickness of 40–50 nm, and (iii) a photoresist layer (As_40_S_40_Se_20_) with a thickness of around 100 nm onto polished glass substrates (F-1 glass, refractive index: *n* = 1.615, dimensions: 20 × 20 × 1 mm) at a residual pressure of 2 × 10^−3^ Pa. The deposition rate and film thickness were monitored in situ with a KIT-1 quartz thickness meter. After deposition, the film thicknesses were measured using a MII-4 microinterferometer.

The recording of periodic structures on photoresist films was carried out using the interference pattern formed with a helium-cadmium laser (wavelength *λ* = 441.6 nm). The exposition value for the gratings recording was 0.2–0.5 J/cm^2^. After exposure, the samples were chemically treated in non-water alkaline organic solutions to form a resistive mask in the photoresist layer, through which the metal film was etched. IL technology was applied in a mode of slight overexposure of the photoresist to provide a cycloid form of the groove profile of the periodic chalcogenide mask. By changing the time for selective etching of the photoresist, it is possible to change the width of the elements of the lithographic masks. Accordingly, the width of the opened intervals between the elements of the mask can be adjusted, through which there is further etching of the metal layer. After removing the photoresist residues in alkaline solution, washing and drying, the metal periodic structure was obtained. Periodic structure was formed only on one half of the chip; the other half remained unetched, i.e., covered with the flat, unstructured gold film. Such samples were prepared for carrying out comparative research using two-channel surface plasmon resonance (SPR) refractometer Plasmon-71 (V. E. Lashkaryov Institute of Semiconductor Physics NAS of Ukraine) with working wavelength of 850 nm, p-polarized radiation (electric vector in the plane of incidence). This device allows measuring the absolute value of reflectivity as well as the absolute value of the angle in the Kretschmann configuration. The wide range of angles for scanning (up to 17° in air) enables quantitative measurements of a reflection curve for liquids with various refraction indexes—from 1.33 (water) up to 1.47 (motor oil) [21].

The surface patterns of the etched structures were examined with a Dimension 3000 Scanning Probe atomic force microscope (Digital Instruments Inc., Tonawanda, NY, USA) in the atomic force microscope (AFM) tapping mode.

## Results and Discussion

The used SPR refractometer Plasmon-71 allows to measure the angular dependences, *R*(*θ*), for the internal light reflection of the gold film and to determine the minimum position of *R*(*θ*) which corresponds to the excitation of the surface plasmon at the interface gold/investigated substance. The minimum position of *R*(*θ*) is very sensitive to changes of the refractive index, *n*, of the environment near the surface of the Au film, which enables recording of processes even for small changes of *n* in this region.

In this work, solutions of glycerol (*n* = 1.474 at 20 °C) in water (*n* = 1.333 at 20 °C) were used for determining the sensitivity of the sensor structures, i.e., the influence of the refractive index of the environment on the shift of the SPR minimum. The solution was introduced into the two-channel flow cuvette, which was located above the sample in a way providing the contact of the investigated liquid with the Au chip. Thus, one channel of the two-channel SPR refractometer Plasmon-71 was responsible for the reference Au film with flat surface, while the second channel recorded the nanostructured film (with the surface relief in the form of the grating).

The spatial frequency of grating was chosen from the proximity condition to Bragg reflection. The theoretical modeling in [15] was carried out for a grating of sinusoidal surface relief with a small relief depth. For such gratings, we can approximately estimate the value of the Bragg period, Λ_B_, using the following equation [22]:

1 $$ {\Lambda}_{\mathrm{B}}=0,5{\lambda}_{\mathrm{o}}\;{\left[\left({\varepsilon}_{\mathrm{mr}}+{\varepsilon}_{\mathrm{D}}\right)/{\varepsilon}_{\mathrm{mr}}{\varepsilon}_{\mathrm{D}}\right]}^{1/2}\;/ \cos \upphi $$

where, *λ*_o_ is the free-space wavelength, *ϕ* is the azimuthal angle—defined as the angle between the plane of incidence and the grating wave vector (that is perpendicular to the grating grooves), *ε*_D_ = *n* ^2^ is the dielectric constant of the investigated dielectric liquid (*n* being the refractive index of this liquid; here: solution of glycerol in water), and *ε*_mr_ is the real part of the dielectric constant of the metal (Au in our investigation).

For an Au/water interface at 850 nm excitation wavelength and *ϕ* = 0, the condition of Bragg resonance according to Eq. (1) corresponds to a grating period near 309 nm (spatial frequency, *ν* = 3240 mm^−1^). Here, we have used the dielectric constant of Au from [23]. Increasing the refractive index of the investigated liquid, the resonance condition is satisfied for lower values of the grating period. Thus, the Bragg resonance for the Au/glycerol interface corresponds to the period of 277 nm (*ν* = 3610 mm^−1^). Therefore, samples were fabricated with spatial periods that fall within this range.

**Fig. 1:**
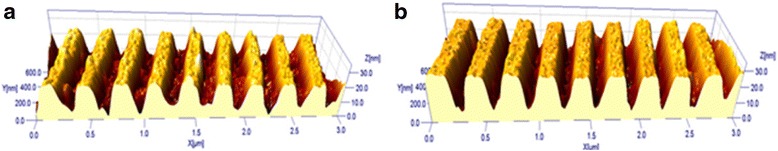
AFM images of Au gratings with a period of 296.6 ± 0.5 nm and average depths of relief of 13.5 ± 2 nm (**a**) and 21.0 ± 2 nm (**b**)

Figure 1 shows, as an example, the AFM images of two gratings with the same period of 296.6 ± 0.5 nm (*ν* = 3372 mm^−1^) but different time of Au etching through the photoresist mask resulting in different average depths of the gratings relief: 13.5 ± 2 nm (a) and 21 ± 2 nm (b).

**Fig. 2:**
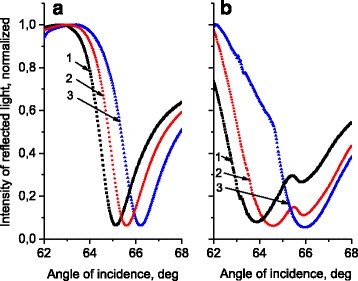
The dependences of the reflection, *R*, on the angle of incidence, *θ*, for the standard Au sensor with flat surface (**a**) and Au gratings with a period of 296.6 ± 0.5 nm and average depth of relief of 13.5 ± 2 nm (**b**). Curve 1 corresponds to *n* = 1.3988, 2: *n* = 1.403, and 3: *n* = 1.4112

Such samples were used for the measurement of the sensor sensitivity. Typical reflection curves (the dependence of the reflectivity, *R*, on the angle of incidence, *θ*) are presented in Fig. 2. Figure 2a shows the reflection curves for the standard sensor with the flat surface of the gold film, which is in contact with the investigated solutions with various refractive indexes. Curve 1 corresponds to *n* = 1.3988, 2: *n* = 1.403, and 3: *n* = 1.4112. As can be seen, the increasing of *n* results in the shift of the position of *R* minimum (position of plasmon resonance), *θ*_min_, toward larger angles; with *n* growing by Δ*n* ≈ 0.012, the value of *θ*_min_ increases by Δ*θ*_min_ = 1.32°. The sensitivity of the method is characterized by the ratio of Δ*θ*_min_ to Δ*n*.

The formation of the periodic grating on the surface of the gold chip changed the shape of the reflection curve in comparison with the same measurements for the standard Au chip. Figure 2b shows the SPR curves for the chip with nanostructured surface; the parameters of the surface relief correspond to Fig. 1a (the grating with depth of relief of 13.5 nm). The sample was fixed in the SPR refractometer so that the plane of incidence of the probing laser beam was parallel to the grating wave vector (perpendicular to the grating grooves). It is seen that the resonance curves are broadened and two minimums are observed. This is consistent with the results of theoretical modeling [15] which showed that for a periodic relief, the SPR curve near Bragg resonance has a two-mode structure with two minima. The shift of the absolute minimum position, Δ*θ*_min_, in the same range of Δ*n* is higher: Δ*θ*_min_ = 2.1°. Hence, an increase in sensitivity for the structured sensor can be noticed as compared to the standard chip.

**Fig. 3:**
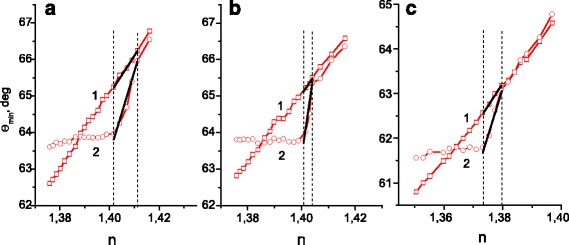
The dependences of the SPR position, *θ*_min_, on the refractive index of the environment, *n*, for Au standard sensors with a flat surface (curve 1 in **a**–**c**) and Au gratings with period of 296.6 ± 0.5 nm and depth of relief of 13.5 ± 2 nm (curve 2 in **a**) and 21.0 ± 2 nm (curve 2 in **b**). Curve 2 in **c** gives the dependence of *θ*_min_ on *n* for the Au grating with period of 302.0 ± 0.5 nm and average depth of relief of 17.5 ± 2 nm

The dependences of the position of the SPR *θ*_min_ value on the refractive index of the environment are shown in more detail in Fig. 3. Curve 1 in all graphs shows the dependence of *θ*_min_ on *n* for the standard Au sensor with unstructured surface. It is seen that with increasing the refractive index *n*, also the angular position of *θ*_min_ increases monotonically and the slope of this dependence (the ratio Δ*θ*_min_ to Δ*n*) is almost constant within the entire investigation interval of *n*—approximately equal to 110 deg./RIU (where RIU is refractive index unit).

The curve 2 in Fig. 3a corresponds to the sensor with the structured surface with small depth of relief (13.5 nm). For the structured sensors, the dependence of SPR position, *θ*_min_, on *n* is nonlinear: by approaching the Bragg resonance, the slope of this dependence is significantly reduced compared to the standard sensor. Consequently, a region with enhanced slope can be observed (and thus with enhanced sensitivity) in the range of the refractive index change Δ*n* = 0.009. In Fig. 3a, this interval is indicated by two vertical dotted lines. The experimental data points in the range of enhanced sensitivity are approximated by segments of straight lines. The ratio of slopes of these segments, i.e., the ratio of the sensitivities of structured and standard sensors in this range of *n*, amounts to 2.4.

Figure 3b (curve 2) shows the same dependences for the structured Au sensor with a greater depth of relief (21 nm). The range of enhanced slope (sensitivity), which is again separated in Fig. 3b by vertical dotted lines, is found to be considerably narrower (Δ*n* = 0.0031) compared with the chip with smaller depth of relief (Fig. 3a), but the value of the slope increased significantly. The ratio of slopes of the segments which approximate the experimental data points for structured and standard sensors within the range of enhanced sensitivity for this sample is equal to 4.4.

It is thus demonstrated that the sensor sensitivity increased from 110 deg./RIU for the standard sensor to 264 deg./RIU for the nanostructured sensor with small depth of relief and up to 484 deg./RIU for the sensor with greater depth of relief. It should be noted that according to theoretical modeling [15], the maximum increase in sensitivity due to formation of the grating reaches 6–6.5. Hence, we explored the potential of real surfaces as compared to ideal surfaces that have been used for the modeling.

The gain in sensitivity of the SPR sensor chips due to gratings, however, is observed in a limited range of refractive index changes. This is also consistent with theoretical results, where enhancement of the sensitivity is predicted in the range of Δ*n* less than 0.01 [15]. Biochemical processes which are investigated by using SPR refractometers are often accompanied by the deposition of monolayers of biomolecules with very small changes in the refractive index values. The refractive index resolution of Plasmon-71 refractometer is Δ*n* = 10^−6^ RIU, so working range Δ*n* even less than 0.01 RIU is sufficient for such measurements.

Furthermore, the working range with enhanced sensitivity of the sensor can be adjusted to the refractive index of the studied environment by changing the spatial frequency of the grating. Figure 3c (curve 2) shows the dependence of the SPR position on the refractive index of the environment for the Au grating with period of 302.0 ± 0.5 nm (spatial frequency, *ν* = 3311 mm^−1^) and average depth of relief of 17.5 ± 2 nm. It is seen, that the interval Δ*n*, where enhancement of the sensitivity is observed, is shifted by 0.026–0.030 RIU to smaller values of *n* in comparison with results for gratings with the period of 296.6 nm (Fig. 3a, b—curves 2). A small variation of the working range positions in Fig. 3a, b can be explained by a slight influence of the depth of relief and the form of the grating grooves. Solving Eq. (1) with respect to *n*, we can approximately estimate the shift of the resonance values of the refractive index due to changes of the grating period from 296.6 to 302 nm. The obtained value for the working range position shift is equal to 0.024 RIU at *ϕ* = 0 and is in good agreement (for this simplified formula) with the above experimental result.

**Fig. 4:**
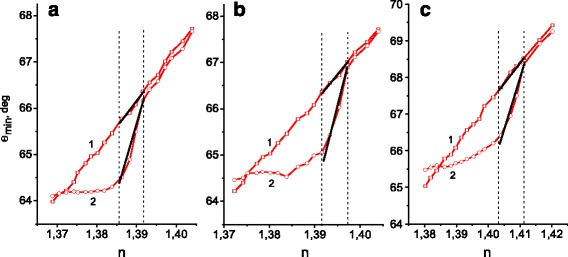
The dependences of SPR position, *θ*_min_, on the refractive index of the environment, *n*, for Au standard sensor (curve 1) and Au gratings with period of 296.6 ± 0.5 nm and depth of relief of 13.5 ± 2 nm (curve 2) coated with a 4-nm chromium layer: **a** the plane of incidence of the probing laser beam is parallel to the grating wave vector (the azimuthal angle *ϕ* = 0), **b** *ϕ* = 5.7°, and **c** *ϕ* = 10°

This possible method of adjusting, however, is rather expensive, because it is necessary to produce a set of structured chips with different spatial frequencies. Importantly, we can also move the working range position by a modification of the chip surface. Figure 4a shows the dependences of the SPR curves’ minimum position on the refractive index of the investigated solution for the Au standard sensor (curve 1) and the Au gratings with depth of relief of 13.5 ± 2 nm (curve 2) coated with a 4-nm thick chromium layer. This island-like Cr layer was deposited by thermal evaporation in vacuum. Such modification of the chip surface results in the shift of the working range position by 0.018 RIU to smaller values of *n* in comparison with non-coated chips (Fig. 3a, curve 2).

Hence, instead of producing gratings with another frequency a thin layer of a metal or a dielectric can be deposited on the surface of the chip. This process facilitates a cheaper grating fabrication. However, this modification of the chip is irreversible and leads to a change in the conditions of immobilization of investigated biomolecules on the chip surface. Therefore, it is intriguing to use a functional ultrathin film, e.g., a plasma polymer film, both for adjusting the working range and the interaction with biomolecules. Such films, however, needs to be highly stable in the used environment in order to maintain the working range, which is currently under investigation [24–26].

The most convenient method for the adjustment of the position for the enhanced sensitivity region with respect to the refractive index of the investigated environment might be a change of azimuth angle. Figure 4b shows the dependences of *θ*_min_ on *n* for the same two-channel chip as in Fig. 4a, but the sample was fixed in the SPR refractometer so that the plane of incidence was rotated by 5.7° with respect to the grating wave vector (*ϕ* = 5.7°). Figure 4c gives the results for the same sample at *ϕ* = 10°. It is evident that the working range was shifted toward higher refractive indices with increasing azimuthal angle, by 0.0056 RIU at *ϕ* = 5.7° and by 0.0184 RIU at *ϕ* = 10°. Estimations by using formula (1) give values for the shift of 0.006 RIU and 0.020 RIU, respectively, which are in good agreement with the experiment. These results demonstrate that with small variations of the azimuth angle, SPR measurements can be performed making use of the increased sensitivity over a wide range of the refractive index change on the single nanostructured chip. This method can also be sued for subsequently coated, structured Au chips.

## Conclusions

The experimental results conducted in this study confirm the predictions of the theory about the potential increase of sensitivity for SPR biosensors by forming a grating with a suitable period and depth of relief on the working surface of the sensors chip. The degree of sensitivity enhancement and the range of the environment refractive index value, in which this enhancement is observed, strongly depend on the depth of the grating relief. The width of the refractive index working range decreases with increasing depth of relief, whereas sensitivity is increased. The working range with enhanced sensitivity of the sensor can be adjusted to the refractive index of the studied environment by changing the spatial frequency of the grating, by modification of the chip surface and most conveniently by rotation of the chip. A two- to fourfold gain in sensitivity can thus be realized for SPR biosensors.

## Abbreviations

AFM: Atomic force microscope
ChG: Chalcogenide glasses
IL: Interference lithography
RIU: Refractive index unit
SPR: Surface plasmon resonance
